# *Acanthamoeba castellanii* : growth on human cell layers reactivates attenuated properties after prolonged axenic culture

**DOI:** 10.1111/j.1574-6968.2009.01680.x

**Published:** 2009-10

**Authors:** Martina Koehsler, David Leitsch, Michael Duchêne, Markus Nagl, Julia Walochnik

**Affiliations:** 1 Department of Medical Parasitology, Clinical Institute of Hygiene and Medical Microbiology, Medical University of ViennaVienna, Austria; 2 Department of Specific Prophylaxis and Tropical Medicine, Center for Physiology, Pathophysiology and Immunology, Medical University of ViennaVienna, Austria; 3 Department of Hygiene, Microbiology and Social Medicine, Division of Hygiene and Medical Microbiology, Innsbruck Medical UniversityInnsbruck, Austria

**Keywords:** *Acanthamoeba*, encystment, protease, axenic culture, *N*-chlorotaurine

## Abstract

The free-living, but potentially pathogenic, bacteriovorous amoebae of the genus *Acanthamoeba* can be easily grown axenically in a laboratory culture. This, however, often leads to considerable losses in virulence, and encystment capacity, and to changes in drug susceptibility. We evaluated potential options for a reactivation of a number of physiological properties, attenuated by prolonged axenic laboratory culture, including encystment potential, protease activity, heat resistance, growth rates and drug susceptibility against *N*-chlorotaurine (NCT). Toward this end, a strain that had been grown axenically for 10 years was repeatedly passaged on human HEp-2 cell monolayers or treated with 5′-azacytidine (AzaC), a methyltransferase inhibitor, and trichostatin A (TSA), a histone deacetylase inhibitor, in order to uplift epigenetic gene regulation. Culture on human cell monolayers resulted in significantly enhanced encystment potentials and protease activities, and higher susceptibility against NCT, whereas the resistance against heat shock was not altered. Treatment with AzaC/TSA resulted in increased encystment rates and protease activities, indicating the participation of epigenetic mechanisms. However, lowered resistances against heat shock indicate that possible stress responses to AzaC/TSA have to be taken into account. Repeated growth on human cell monolayers appears to be a potential method to reactivate attenuated characteristics in *Acanthamoeba*.

## Introduction

The primary free-living acanthamoebae have been serving as suitable model organisms for studies on cell biology for decades, due to their rapid growth, relatively large size and low nutritional requirements (Schuster, 2002). Since the early 1970s, they have also been recognized as facultative pathogens causing granulomatous amoebic encephalitis, a fatal condition (Marciano-Cabral & Cabral, 2003), in immunocompromised persons, and *Acanthamoeba* keratitis (AK) (Illingworth & Cook, 1998) in otherwise healthy individuals. To date, it is still not entirely understood what determines the pathogenic potential of *Acanthamoeba* strains, but several physiological properties are likely to play a role.

Rapid encystment and high protease activity can be considered important traits of free-living acanthamoebae, ensuring survival under changing environmental conditions and providing the basis for nutrition, respectively. During the course of an infection, these two properties work similarly. Amoebae encyst to escape therapeutic measures or the immune response and secrete proteases to invade tissues, concomitantly digesting human cells (Martinez & Visvesvara, 1997; Clarke & Niederkorn, 2006;).

Research on *Acanthamoeba*'s pathogenic potential is complicated by its tendency to downregulate virulence and encystment capacity and to display altered drug sensibilities upon prolonged axenic laboratory culture (Mazur & Hadas, 1994; Hughes *et al.*, 2003; Koehsler *et al.*, 2008). These adaptations clearly influence studies on the pathogenic potential and drug susceptibility of *Acanthamoeba*, and an effective method to maintain or reactivate properties of freshly isolated strains is highly desirable.

It has been shown that a decrease in the virulence of acanthamoebae is not permanent and can be restored by multiple mouse passages (Mazur & Hadas, 1994). Also, for *Naegleria fowleri*, serial mouse passage has been used for the restoration of virulence; however, a considerable variation in the response of mice to the amoebae has been observed (Cursons & Brown, 1978). An alternative method was described by John & John (1994), who demonstrated a virulence-enhancing effect in *N. fowleri* after repeated growth on cell cultures, while in *Acanthamoeba* growth on cell monolayers has been serving as an indicator for the pathogenic potential of strains (Cursons & Brown, 1978; Walochnik *et al.*, 2000;).

The fact that loss of virulence of *Acanthamoeba* is not necessarily permanent and that encystment capacity decreases as early as within the first 6 months in an axenic culture (Mazur & Hadas, 1994; Koehsler *et al.*, 2008;) very much argues for an involvement of epigenetic modifications. These are believed to be environmentally stimulated and include DNA methylation and/or histone modifications, resulting in gene silencing and/or increased expression of genes (Moazed, 2001; Peterson & Laniel, 2004;). 5′-Azacytidine (AzaC), a methyltransferase inhibitor, and trichostatin A (TSA), a histone deacetylase inhibitor, have already been successfully used in a variety of organisms to abrogate epigenetic regulation in a selective manner (Bernes *et al.*, 2005; Isakov *et al.*, 2008;).

In order to elucidate the probable mechanisms involved in adaptations to axenic culture and to evaluate possible *in vitro* options to reactivate attenuated properties of *Acanthamoeba* strains, the impact of cell–cell contact with human Hep-2 cells and of treatment with AzaC and TSA on an *Acanthamoeba castellanii* strain (1BU) that had been axenically cultured for 10 years was determined with regard to its protease activity and encystment capacity. Additionally, the impact on growth rates, heat resistances and susceptibility against *N*-chlorotaurine (NCT) was evaluated. NCT, the *N*-chloro derivative of the amino acid taurine, is a long-lived oxidant produced by human granulocytes and monocytes from hypochlorite and taurine during the oxidative burst. It can be used as an antiseptic in different body regions, and its amoebicidal activity has been demonstrated recently (Fuernkranz *et al.*, 2008).

## Materials and methods

### Culture of 1BU, 1BUAT and 1BUHEp3x

*Acanthamoeba castellanii* strain 1BU (ATCC no. PRA-105), genotype T4, isolated from a corneal specimen of a keratitis patient in 1998, and grown axenically since then, was used in this study (Walochnik *et al.*, 2000). This strain was cultivated under three different conditions: (1) axenically in proteose peptone–yeast extract–glucose medium (PYG) (Visvesvara & Balamuth, 1975) at 26 °C (referred to as 1BU in the following experiments); (2) in PYG in the presence of AzaC (23 μM) and TSA (70 nM) for 72 h (referred to as 1BUAT); and (3) three times consecutively on HEp-2 monolayers (referred to as 1BUHEp3x) as described previously (Walochnik *et al.*, 2000). In brief, HEp-2 cells were cultured in a 1 : 1 mixture of PC-1 (Bio-Whittaker, Walkersville, MD) and CO_2_-independent medium (Life Technologies Ltd, Paisley, Scotland) supplemented with l-glutamine (2 mM) in 150-cm^2^ tissue culture flasks at 37 °C under sterile conditions until the monolayer covered the bottom of the flask completely. Amoebae (10^5^) suspended in 25 mL physiological NaCl (0.9%) were inoculated onto the monolayer, three times consecutively. Cocultures of amoebae and tissue cells were incubated at 26 °C until the monolayer was completely lysed. For all experiments, 1BU, 1BUAT and 1BUHEp3x were transferred into fresh PYG *c*. 18 h before experiments.

### Growth rates/generation times

1BU, 1BUAT and 1BUHEp3x, respectively, were transferred into fresh PYG, starting with 10^4^ cells mL^−1^. Cells were counted daily until the stationary phase was reached. Growth rates and generation times were calculated.

### Induction of encystment and differential cell counts

Trophozoites (5 × 10^5^ mL^−1^) of 1BU, 1BUAT and 1BUHEp3x were induced to encyst using a Tris-buffered medium (95 mM NaCl, 5 mM KCl, 8 mM MgSO_4_, 0.4 mM CaCl_2_, 1 mM NaHCO_3_ and 20 mM Tris-HCl, pH 9.0) as described previously (Koehsler *et al.*, 2008). Additionally, encystment potentials of 1BU after one and five growth passages on HEp-2 cells were evaluated. Progress of encystment was quantified using a Fuchs–Rosenthal counting chamber at the beginning and 24, 48 and 72 h after induction of encystment, respectively.

### Zymography

Somatic and excreted proteases of 2 × 10^6^ cells per assay were prepared and gel electrophoresis of 10 μL diluted sample (lysates 1 : 50, supernatants 1 : 10) was carried out on 12% sodium dodecyl sulfate polyacrylamide gels containing 0.2% gelatin at 25 mA and 4 °C as described previously (Blaschitz *et al.*, 2006). Additionally, samples were treated with the serine protease inhibitor phenylmethylsulfonyl fluoride (PMSF) (2 mM), the cysteine proteinase inhibitor E-64 (2 μM) and the metalloproteinase inhibitor 1,10-phenanthroline (10 mM) 45 min before electrophoresis. For inhibition experiments 1,10-phenanthroline, a reversible inhibitor, was also added to the developing buffer. Additionally, to confirm the results of zymography experiments, 10^5^ amoebae of 1BU, 1BUAT and 1BUHEp3x were inoculated onto HEp-2 monolayers as described above, and after 48 h, progress of monolayer lysis was evaluated by light microscopy and compared.

### Resistance against heat shock

1BU, 1BUAT and 1BUHEp3x were exposed to a variety of temperatures (40, 42, 45 and 48 °C) for 1 h. Subsequently, the viability of trophozoites was determined by phase-contrast microscopy and trypan blue staining. According to our observations, exposure to 42 °C was the most suitable condition for the assay because higher temperatures rapidly decreased cell viability and lower temperatures resulted in only a slight effect. Based on these observations, 1BU was exposed to heat shock at 42 °C in PYG for 1 h and subsequently grown in fresh PYG for 18 h. Amoebae were induced to encyst, and protease activities were evaluated as described above with untreated strain 1BU as a control.

### Drug susceptibility tests

Susceptibility tests against NCT were carried out in 24-well microtiter plates. Amoebae (10^5^) of 1BU, 1BUAT and 1BUHEp3x, respectively, were resuspended in 1 mL PYG, and 1 M NCT in H_2_O was added to reach final concentrations of 15 and 30 mM NCT. After 2, 6 and 24 h, dead and living cells were counted in a Fuchs–Rosenthal chamber by phase-contrast microscopy and trypan blue staining. Percentages of dead cells were calculated. 1BU, 1BUAT and 1BUHEp3x without NCT were used as growth controls.

### Statistics

All experiments were carried out in three independent setups with triplicate samples. Mean values and SDs were calculated. For encystment and drug susceptibility experiments, single-factor anova and Tukey's HSD (spss, version 14.0) were used to test for statistical significance, and *P*-values <0.05 were considered significant.

## Results

### Growth rates

Generation times refer to the average time between two cell divisions of *Acanthamoeba* trophozoites. 1BU, grown axenically in PYG, exhibited the highest growth rate and the shortest generation time with 26.3 h. 1BUHEp3x, coincubated three times with HEp-2 monolayers, exhibited a generation time of 29.8 h, while 1BUAT, grown in the presence of AzaC and TSA, exhibited the lowest growth rate, resulting in a generation time of 34.8 h.

### Encystment rates are increased by HEp-2 cell passage and AzaC/TSA treatment

**Fig. 1:**
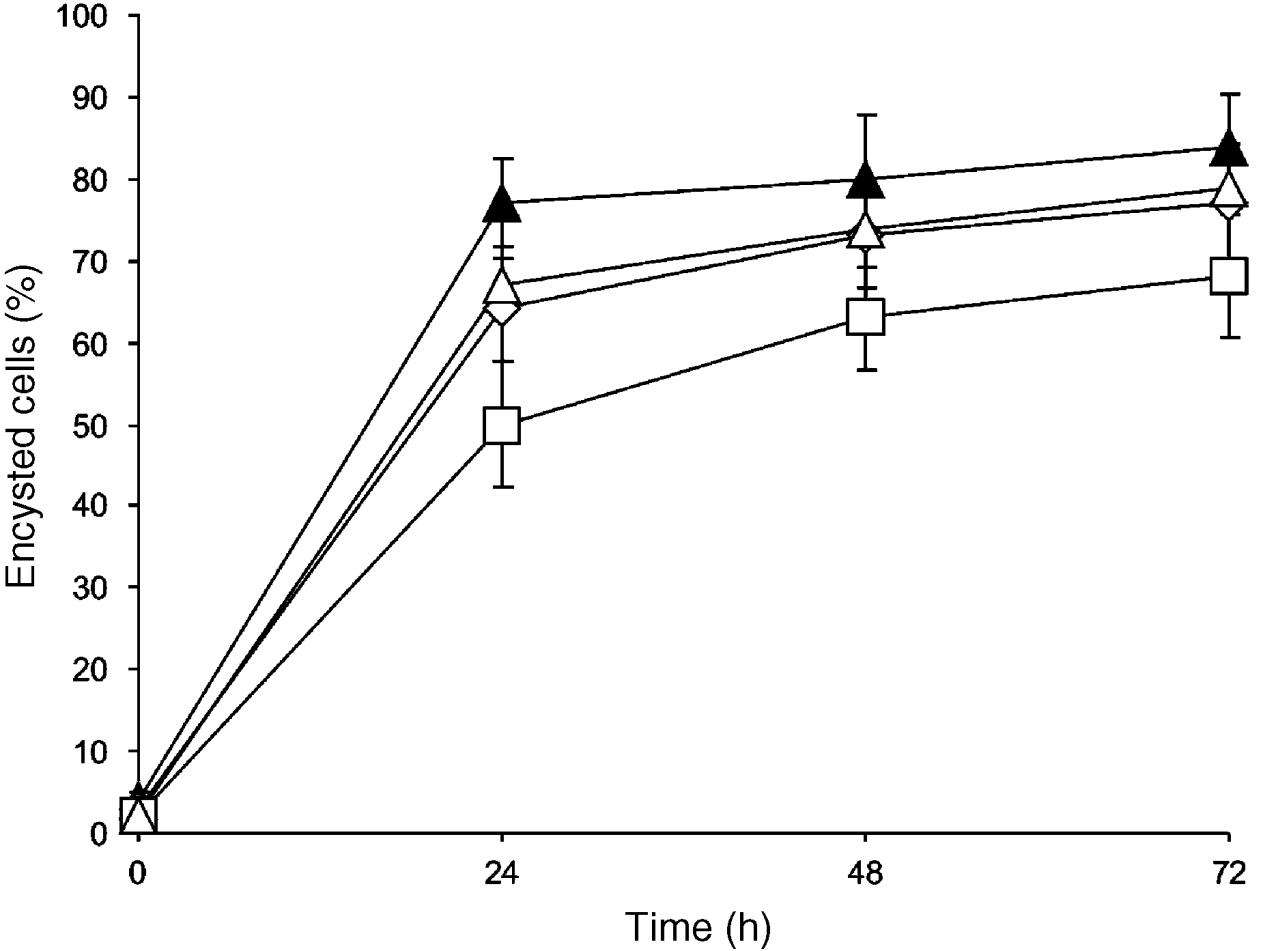
Progress of encystment of 1BU (□), grown in PYG; 1BUAT (◊), grown in the presence of AzaC/TSA; 1BUHEp1x (▵), grown one time on HEp-2-monolayers; and 1BUHEp3x (▴), grown three times consecutively on HEp-2-monolayers, in percent. 1BUHep1x, 1BUHep3x and 1BUAT exhibited significantly higher cyst rates (*P*<0.001).

Percentages of encysted cells of 1BU, 1BUAT and 1BUHEp3x are shown in Fig. 1. Also included are the results obtained with 1BU after a single cycle of coincubation with HEp-2 cells (1BUHEp1x). AzaC/TSA treatment as well as Hep-2 contact led to increased cyst rates in strain 1BU. This effect was most pronounced after 24 h, with an increase of 14% of cysts for 1BUAT, 17% for 1BUHEp1x and 27% for 1BUHEp3x, respectively (*P*<0.001). In 1BUHEp3x, synchronous encystment, which is defined as >70% cysts after 24 h, was recovered (Byers, 1979). Even after only one passage on HEp-2 cells, cyst rates were higher than after treatment with AzaC/TSA (*P*<0.001). Interestingly, five passages on HEp-2 cells did not result in higher cyst rates than three passages (data not shown). Apparently, there is an upper limit for the reactivation of the encystment capacity, which cannot be further enhanced by additional passages on cell monolayers.

### Increased protease activity after HEp-2 cell passage and AzaC/TSA treatment

**Fig. 2:**
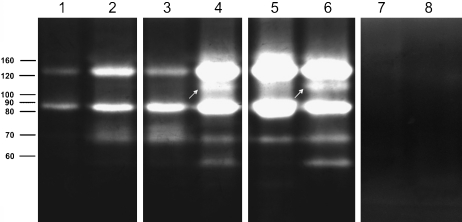
Somatic and excreted proteases of 2 × 10^6^ cells of 1BU, grown in PYG, 1BUAT, grown in the presence of AzaC/TSA, and 1BUHEp3x, grown three times consecutively on HEp-2-monolayers on a 12% PAA-gel containing 0.2% gelatine. Lane 1, 1BU lysate diluted 1 : 50; lane 2, 1BU supernatant diluted 1 : 10; lane 3, 1BUAT lysate diluted 1 : 50; lane 4, 1BUAT supernatant diluted 1 : 10; lane 5, 1BUHEp3x lysate, diluted 1 : 50; lane 6, 1BUHEp3x supernatant, diluted 1 : 10; lane 7, 1BUHEp3x lysate, diluted 1 : 50, +2 mM PMSF; and lane 8, 1BUHEp3x+2 mM PMSF supernatant, diluted 1 : 10. Marker lane in kDa. White arrows indicate a new protease band at an approximate weight of 107 kDa.

AzaC/TSA treatment and HEp-2 contact resulted in increased protease activities of whole-cell lysates as well as of supernatants (Fig. 2). In 1BUHEp3x, protease activity of whole-cell lysates and the supernatants was significantly stronger, while in 1BUAT, the increase of activity was most pronounced in the supernatant. Increases could be observed at a molecular weight of *c*. 130, 85 and 55 kDa in the supernatants and at 130 and 85 kDa in the whole-cell lysates. At a weight of *c*. 107 kDa, a band appeared in the supernatants, which might be expressed too weakly to be visible in 1BU. All proteases were identified as putative serine proteases, because treatment with PMSF (2 mM) resulted in complete inhibition of activity (Fig. 2), while E-64 and 1,10-phenanthroline did not lead to any inhibition (data not shown). These results are corroborated by the observed cytopathic activity when grown on an HEp-2 monolayer for 48 h. 1BUHEp3x and 1BUAT lysed the monolayer completely, while only an estimated 70% of the monolayer was lysed by 1BU.

### Influence of treatments on heat-shock resistance

**Fig. 3:**
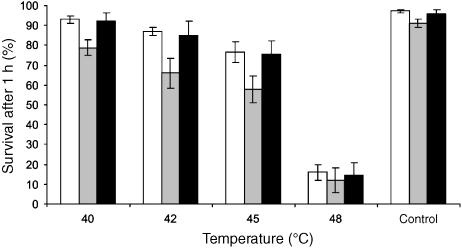
Percentage of surviving cells after exposure to heat shock at different temperatures for 1 h of 1BU, grown in PYG (white columns); 1BUAT, grown in the presence of AzaC/TSA (grey columns); and 1BUHEp3x, grown three times consecutively on HEp-2-monolayers (black columns).

1BU and 1BUHEp3x were similarly resistant to heat shock, while 1BUAT was less resistant in all experiments. Heat shock at 42 and 45 °C led to the highest differences in resistances, with 18–21% more dead cells in 1BUAT (Fig. 3). For encystment and protease activity experiments, heat shock at 42 °C (survival rate of strain 1BU 86.8%) was chosen, which is sufficiently ‘shocking’ the cells, but only killing a small percentage. After 1 h, most cells had rounded up and had detached from the bottom of the flask. Surviving cells regained their regular shape and reattached to the bottom of the flask within a few hours under standard conditions, while dead cells remained floating in the medium and were discarded.

### The previously observed changes are due to a specific effect of HEp-2 contact and exposure to AzaC/TSA

In order to elucidate whether the previously observed activation of encystment potential and protease expression was due to a specific effect of HEp-2 contact and AzaC/TSA exposure rather than a result of cellular stress caused by these treatments, cells were heat shocked as described above. Subsequently, encystment was induced and protease activities were determined. Encystment rates were very similar at all times, with only *c*. 2% of deviation from the untreated control. Furthermore, no visible changes in protease activities could be observed. These results indicate that increases in encystment potential and protease activities after AzaC/TSA treatment and HEp-2 contact are not due to a regular stress response.

### Susceptibility to NCT

**Fig. 4:**
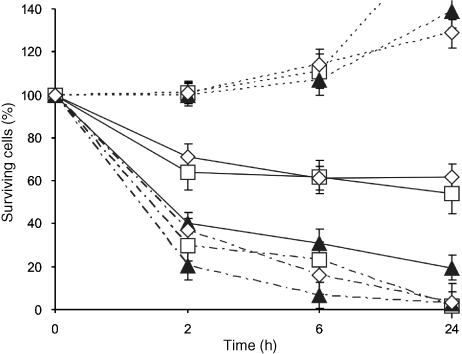
Susceptibility to NCT of 1BU, grown in PYG (□); 1BUAT, grown in the presence of AzaC/TSA (⋄); 1BUHEp3x, grown three times consecutively on HEp-2-monolayers (▴), at a concentration of 15 mM NCT (solid line), 30 mM NCT (solid-dashed line), untreated control (dashed line) after 2, 6 and 24 h. 1BUHep3x was significantly more susceptible than 1BU and 1BUAT at both concentrations after 2 and 6 h (*P*<0.001).

The susceptibilities of 1BU, 1BUAT and 1BUHEp3x to NCT are shown in Fig. 4. At a concentration of 15 mM NCT, 1BUHEp3x was significantly more susceptible than 1BU and 1BUAT, with *c*. 50% more dead cells at all times (*P*<0.001). After 2 h, there were also notable differences between 1BUAT and 1BU (*P*<0.001) to reach similar levels after 6 h until the end of incubation. At a concentration of 30 mM NCT, 1BUHEp3x was also more susceptible after 2 and 6 h, but this concentration was lethal for most cells of all three strains after 24 h and no significant differences in susceptibility could be detected (*P*<0.5). In general, the reaction to NCT was most pronounced within the first 2 h of incubation. Because the oxidant NCT is inactivated mainly by thio groups, consumption of NCT by reaction with medium or cell components has to be taken into consideration (Fuernkranz *et al.*, 2008).

## Discussion

Adaptations to axenic laboratory conditions are a common phenomenon and have been reported for several protozoa (Wong *et al.*, 1977; Segovia *et al.*, 1992;). Loss of virulence is most apparent, but loss of encystment capacity has also been described (Phillips, 1973; Koehsler *et al.*, 2008;). It is conceivable that under laboratory conditions, under which an optimal environment and abundant nutritive supply are provided, energy-consuming properties such as virulence and encystment might be the first to be ‘discarded’ in order to achieve elevated growth rates. Indeed, the observed growth rates in our study corroborate this hypothesis as untreated 1BU exhibited the highest growth rates, while 1BUHEp3x and 1BUAT showed longer generation times. Another example for adaptation to axenic culture resulting in optimization of growth are *Leishmania* spp., which become more efficient in utilizing biopterin, an important growth factor, due to the overexpression of several genes after several passages (Roy *et al.*, 2001).

Both cell contact and AzaC/TSA treatment led to significantly increased encystment rates compared with 1BU (*P*<0.001), and cell contact was considerably more effective in this regard. Yang & Villemez (1994) demonstrated that encystment of *Acanthamoeba* is receptor-mediated and that antibodies inducing encystment concomitantly inhibit pinocytosis, indicating that a lack of nutrients might be a major stimulus for encystment. Thus, when grown on HEp-2 layers without additional nutrients, amoebae might sense the increasing nutritive loss, and initiate mechanisms to be prepared for evasion.

Phagocytosis plays an important role in the pathogenesis of *Acanthamoeba* (Niederkorn *et al.*, 1999), indicating that nutrition might also be an important trigger, for protease activity. This is corroborated by our findings because amoebae, grown on cell monolayers as a substrate, exhibited highly elevated protease activities, even more than amoebae after treatment with AzaC/TSA. Nevertheless, 1BUAT showed an identical protease pattern and increases of the same proteases. The increase of protease activity was reflected by higher cytopathic effects on HEp-2 monolayers. Upon isolation from a corneal sample of an AK patient, strain 1BU exhibited a strong cytopathic effect (scored ++ on a + to +++ scale), corresponding to 75% lysis of a cell monolayer (Walochnik *et al.*, 2000). In the current study, the intrinsic cytopathogenic effect of 1BU had slightly declined (to 70%), but could be enhanced to 100% by HEp-2 contact and also by AzaC/TSA treatment. Apart from the observed higher cytopathic effects of both strains compared with 1BU, increases in virulence are particularly indicated by a higher expression of the secreted 130-kDa protease, which might represent a mannose-induced protease with a weight of 133 kDa (MIP-133) that is clearly associated with pathogenicity (Hurt *et al.*, 2003). The newly expressed secreted protease with an approximate weight of 107 kDa might resemble a previously described protease that is expressed only in pathogenic isolates as described by Khan *et al.* (2000).

The results for encystment and protease activities indicate a connection between these two properties, because both were enhanced by treatment with AzaC/TSA and HEp-2 contact. This notion is supported by recent findings, because a 33-kDa serine protease has been shown to be involved in the encystment process of *Acanthamoeba* (Moon *et al.*, 2008). Moreover, inhibition of serine protease activity by PMSF or siRNA-targeting serine proteinase genes resulted in significant loss of encystment and excystment capabilities of *Acanthamoeba* (Dudley *et al.*, 2008). An involvement of proteases in encystment has also been reported for *Entamoeba invadens* and *Giardia lamblia* (Riahi & Ankri, 2000; Dubois *et al.*, 2008;).

Heat shock experiments were undertaken to investigate whether regular stress has a similar effect on encystment potential and protease activity as has HEp-2 contact and AzaC/TSA treatment. Our results demonstrate that this is not the case, as heat-shocked trophozoites of strain 1BU did not exhibit changed encystment rates and protease activities. 1BUHEp3x was similarly resistant against heat shock as 1BU, indicating that the stress response is not altered by growth on HEp-2 monolayers. Treatment with AzaC/TSA resulted in a reduced tolerance to heat shock, which could be due to the toxic effects of AzaC/TSA that compromise the robustness of the cell and lower the resistance to additional stress.

Another interesting aspect was the impact of HEp-2 contact and AzaC/TSA treatment on the susceptibility against NCT. Obviously, HEp-2 contact enhances the susceptibility against NCT, which was highly pronounced at a concentration of 15 mM (*P*<0.001). This could be due to the high rate of phagocytosis required to digest HEp-2 monolayers. Noble *et al.* (2002) reported an increased susceptibility of *Acanthamoeba* spp. against polyhexamethylene biguanide in the presence of yeast and bacteria they could phagocytose. This was explained by alterations of the cytoplasmic membrane that enhanced pinocytosis, but the direct uptake of drugs with phagocytosis seems to be another plausible explanation. In contrast, treatment with AzaC/TSA did not alter the susceptibility against NCT, suggesting that it requires cell-to-cell contact for the activation of phagocytosis-specific genes. In conclusion, HEp-2 passage proved to be an effective method to increase the protease activity and encystment potential of *Acanthamoeba* after a prolonged axenic culture. This finding might facilitate further research on this facultative pathogen. Finally, an involvement of the epigenetic regulation of gene expression in the loss and regain of physiological properties in *Acanthamoeba* was demonstrated. However, whether this is a general mechanism in *Acanthamoeba* has to be clarified using more strains.

## Statement

Re-use of this article is permitted in accordance with the Terms and Conditions set out at http://www3.interscience.wiley.com/authorresources/onlineopen.html
